# Decreased BOLD signals elicited by 40-Hz auditory stimulation of the right primary auditory cortex in bipolar disorder: An fMRI study

**DOI:** 10.3389/fpsyt.2022.833896

**Published:** 2022-09-15

**Authors:** Hiroshi Okamoto, Toshiaki Onitsuka, Hironori Kuga, Naoya Oribe, Naho Nakayama, Shou Fukushima, Tomohiro Nakao, Takefumi Ueno

**Affiliations:** ^1^Division of Clinical Research, National Hospital Organization, Hizen Psychiatric Medical Center, Saga, Japan; ^2^Department of Neuropsychiatry, Graduate School of Medical Sciences, Kyushu University, Fukuoka, Japan; ^3^Department of Neuroimaging Psychiatry, Graduate School of Medical Sciences, Kyushu University, Fukuoka, Japan; ^4^National Center for Cognitive Behavioral Therapy and Research, National Center of Neurology and Psychiatry, Tokyo, Japan; ^5^Medical Corporation Kouseikai, Michinoo Hospital, Nagasaki, Japan

**Keywords:** bipolar disorder, mood disorder, BOLD, fMRI, neuroimaging, clinical neurophysiology, auditory steady-state response, gamma oscillations

## Abstract

**Background:**

A number studies have been conducted on abnormalities in the cortical circuitry of gamma oscillations, including deficit in auditory steady-state response (ASSR) to gamma-frequency (≧ 30-Hz) stimulation, in patients with bipolar disorder (BD). In the current study, we investigated neural responses during click stimulation by blood oxygen level-dependent (BOLD) signals. We focused on Broadman 41 and 42, the main sources of ASSR.

**Materials and methods:**

We acquired BOLD responses elicited by click trains of 80-, 40-, 30- and 20-Hz frequencies from 25 patients with BD to 27 healthy controls (HC) with normal hearing between 22 and 59 years of age assessed *via* a standard general linear-model-based analysis. We extracted contrast values by identifying the primary auditory cortex and Brodmann areas 41 and 42 as regions of interest (ROI)s.

**Results:**

BD group showed significantly decreased ASSR-BOLD signals in response to 40-Hz stimuli compared to the HC group in the right Brodmann areas 41 and 42. We found significant negative correlations between the BOLD change in the right Brodmann areas 41 and 42 and Structured Interview Guide for the Hamilton Depression Rating Scale (SIGH-D) scores, also the BOLD change in the right Brodmann areas 41 and 42 and the Positive and Negative Syndrome Scale (PANSS)-Negative scores.

**Conclusion:**

The observed decrease in BOLD signal patterns in the right primary auditory cortex during 40-Hz ASSR may be a potential biomarker option for bipolar disorder.

## Introduction

Auditory steady-state response (ASSR) is a periodic electrical response from the brain evoked by auditory click stimuli synchronized to both the frequency and the phase of the click stimulus. Responses between 14 and 30 Hz are classified as beta-band activity, while rhythms ≧ 30 Hz are classified as gamma-band activity (1). Furthermore, gamma band activity is subclassified into low gamma band (30–70 Hz) and high gamma band (above 70 Hz) oscillations (2). Previous studies using magnetoencephalography (MEG) showed that the source generators of ASSR are localized to the primary auditory cortex (3, 4). Some think that it is unclear whether the conclusion of their studies, which states that gamma oscillations originate in the auditory cortex, is accurate. However, it’s correct that in general, gamma oscillations are thought to be generated at the local microcircuit level (5, 6). Therefore, it would not be surprising that the auditory cortex generates its own gamma rhythm. The resonant frequencies of the ASSR are approximately 40-Hz and 80-Hz in healthy individuals, and larger power is observed at 40-Hz (7). At these frequencies, the ASSR power is augmented. The mechanism for the generation and maintenance of gamma oscillations at the cellular level relies on a network of fast-spiking parvalbumin-expressing gamma aminobutyric acid (GABA)-ergic interneurons (8). The ASSR has been suggested to reflect the activity of efficient GABA inhibitory interneurons that control the timing of pyramidal cells in layer II/III of the cortex (9, 10). In addition, the interaction of pyramidal cells with inhibitory neurons has been shown to generate emergent oscillations (11). Moreover, the amplitude and phase of the ASSR are considered to reflect the balance between inhibitory GABAergic activity and excitatory glutamatergic activity *via N*-methyl-*D*-aspartate (NMDA) receptors (12–14).

In a postmortem study, it has been reported that BD patients show the down-regulated expression of GABAergic genes such as glutamic acid decarboxylase (15). Moreover, NMDA receptor dysfunction, which causes preferential disruption of GABAergic circuits, considered to contribute to the pathophysiology of BD (16–20). In the more recent study, Sherif et al. (21) examined the effects of alterations in NMDA receptor, hyperpolarization-activated cyclic nucleotide-gated (HCN) channel that codes for Ih current, and GABA_*A*_ receptor on information flow, and in gamma activity in local field potential (LFP) using a hippocampal CA3 computer model with 1,200 neurons. They found that altering NMDA receptor, GABA_*A*_ receptor, Ih, individually or in combination, modified information flow in an inverted-U shape manner, with information flow reduced at low and high levels of these parameters. Theta-gamma phase-amplitude coupling also had an inverted-U shape relationship with NMDA receptor augmentation. The strong information flow was associated with an intermediate level of synchrony, seen as an intermediate level of gamma activity in the LFP, and an intermediate level of pyramidal cell excitability. Their results were consistent with the idea that overly low or high gamma power is associated with pathological information flow and information processing.

The investigation of gamma band ASSR is important for understanding BD because gamma band ASSR is associated with an imbalance between GABAergic and NMDA receptor-mediated neurotransmission. In studies using MEG, Maharajh et al. (22) reported that BD patients exhibited a reduced right 40-Hz ASSR and Oda et al. (23) reported that BD patients exhibited a reduced bilaterally 80-, 40-, and 30-Hz ASSR. In a study using electroencephalography (EEG), it has been reported that BD patients exhibit 50-, 40-, 30-, and 20-Hz ASSR reduced (24). Moreover, it has been reported that BD patient’s exhibit reduced ASSR synchronization at 50- and 40-Hz stimulation and reduced ASSR power at 40 Hz (25).

A number of studies have consistently reported that gamma band ASSR is reduced in schizophrenia (26–31). Thus, patients with BD and SZ have been found to have similar patterns of ASSR abnormalities. Further, a decrease in the number density of inhibitory interneurons in both SZ and BD was reported in a post-mortem study (32). These findings suggest that there is some degree of common dysfunction of neural circuits in these diseases. As a whole, there is less attention paid to ASSR in BD research compared to SZ research.

**FIGURE 1:**
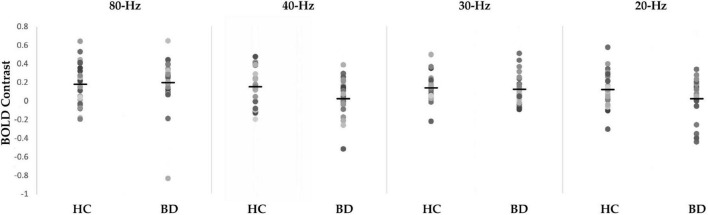
Scattergrams of the blood oxygen level-dependent (BOLD) contrast of 80-, 40-, 30-, and 20-Hz auditory steady-state response stimulation (ASSR) between healthy controls (HC) group and bipolar disorder (BD) group in the right Brodmann areas 41 and 42. The BD group showed significantly decreased ASSR-BOLD signals to the 40-Hz stimuli compared with HC group (*p* = 0.029). Horizontal lines indicate group means.

Most ASSR studies have been conducted with EEG or MEG, however it was found that there is a strong correlation between hemodynamic signals and synchronized gamma oscillations (33). In the feline visual cortex, hemodynamic changes have been reported to be significantly and positively correlated with neuronal synchronization in the gamma range (between 52 and 90 Hz) (33). Furthermore, it has been reported that specific gamma-blood oxygen level-dependent (BOLD) correlations are found during a visual attention task in humans (34). Other studies have consistently shown that gamma oscillations play a major role in neurovascular coupling (35–40). Moreover, neural oscillations in the gamma band have been reported to be particularly associated with increased mitochondrial oxidative metabolism, characterized by higher oxygen consumption and mitochondrial gene expression, which suggests a significant association between neural oscillations in the gamma band and BOLD signal (41). Accordingly, functional magnetic resonance imaging (fMRI), may be suited to evaluate evoked gamma and spontaneous gamma oscillations during periodic click stimulation with BOLD signals. The relationships between fMRI signals and electrophysiological responses for ASSR were shown in “Figure 1” of our previous article (42). Our group reported that the acute episode schizophrenia group showed significantly increased ASSR-BOLD signals to 80-Hz stimuli in the left auditory cortex compared to the healthy controls and non-acute episode schizophrenia groups (42). Nevertheless, no study has examined ASSR-BOLD signals in patients with BD to our knowledge.

In this study, we examined high gamma (ASSR to 80 Hz click trains), low gamma (ASSR to 40 and 30 Hz click trains) and beta (ASSR to 20 Hz click trains) ASSR-BOLD signals in healthy controls (HC), patients with bipolar disorder (BD). We investigated whether ASSR-BOLD signals would be altered in the BD group. Based on the previous ASSR studies using EEG and MEG, we hypothesized that BOLD signals elicited by the gamma frequency periodic stimuli would be decreased in the BD group.

## Materials and methods

This study was conducted according to the guidelines of the Declaration of Helsinki, and approved by the Ethics Committee of National Hospital Organization, Hizen Psychiatric Medical Center (approval number: 25-7, date: July 25, 2013).

### Subjects

Table 1 shows demographic and clinical data. The sample was composed of 27 HC, 25 BD subjects. All subjects had normal hearing, were between 22 and 59 years of age. 51 subjects were right-handed and 1 BD subject was left-handed (43). After receiving a complete explanation of this study, all participants signed an informed consent form in accordance with the regulations of the Ethics Committee of the National Hospital Organization Hizen Psychiatric Medical Center. Healthy controls were screened using the Structured Clinical Interview (SCID)-non-patient edition (44). Healthy controls or their first-degree relatives did not have an Axis-I psychiatric disorder. The exclusion criteria were: (1) a verbal intelligence quotient below 75, (2) previous treatment with electroconvulsive therapy, (3) neurological illness or major head trauma, (4) alcohol or drug dependence, and (5) alcohol or drug abuse within the past 5 years.

**TABLE 1:** Demographic and clinical characteristics of the study groups.

	HC	BD	*t* or *χ^2^*	df	*P*-value
Age (years)	± 11.2	± 11.1	−0.90	50	0.37
Sex (male), N (%)	14 (52)	9 (36)	1.32	1	0.25
WAIS-III information subscale	9.9 ± 2.7	8.5 ± 3.2	1.75	49	0.086
SES	± 0.6	2.8 ± 1.1	−2.79	50	0.007
Handedness	± 7.2	92.6 ± 29.4	0.87	50	0.39
Onset age (years)	/	35.6 ± 11.8			
Duration of illness (months)	/	76.4 ± 84.9			
PANSS-total	/	29.5 ± 8.0			
PANSS-positive	/	13.1 ± 5.4			
PANSS-negative	/	10.9 ± 3.7			
BPRS-total	/	43.7 ± 12.2			
YMRS-total	/	9.0 ± 8.5			
SIGH-D	/	8.6 ± 5.9			
Chlorpromazine equivalents (mg)	/	384.6 ± 217.4			

Values are mean ± SD unless otherwise noted. HC, healthy controls; BD, bipolar disorder; SES, socioeconomic status; WAIS-III, Wechsler Adult Intelligence Scale-Third Edition; PANSS, positive and negative syndrome scale; BPRS, brief psychiatric rating scale; YMRS, Young mania rating scale; SIGH-D, structured interview guide for the Hamilton depression rating scale. 19 BD Patients were medicated with mood stabilizers (N = 9 lithium; N = 4 valproate; N = 1 carbamazepine; N = 5 lithium and valproate), 18 BD patients were medicated with antipsychotics (N = 6 olanzapine; N = 2 quetiapine; N = 2 aripiprazole; N = 2 risperidone; N = 1 haloperidol; N = 2 quetiapine and aripiprazole; N = 1 quetiapine and risperidone; N = 1 olanzapine and chlorpromazine; N = 1 quetiapine, chlorpromazine and levomepromazine), 6 BD patients received additional medications with antidepressants (N = 2 sertraline; N = 1 fluvoxamine; N = 1 duloxetine; N = 1 venlafaxine; N = 1 duloxetine and trazodone), 10 BD patients received additional medications with benzodiazepines (N = 5 flunitrazepam; N = 1 brotizolam; N = 1 etizolam; N = 2 flunitrazepam and brotizolam; N = 1 flunitrazepam and etizolam).

We recruited all patients from National Hospital Organization Hizen Psychiatric Medical Center, and they were diagnosed based on the SCID-DSM IV-TR, Research Version (45) and information from patient medical records. 3 BD participants were outpatients, and 22 BD participants were inpatients. The patients were assessed using Young Mania Rating Scale (YMRS) (46), Structured Interview Guide for the Hamilton Depression Rating Scale (SIGH-D) (47), the Positive and Negative Syndrome Scale (PANSS) (48), and the Brief Psychiatric Rating Scale (BPRS) (49).

### Stimuli and procedure

After a hearing test, we asked participants to lay supine inside an MRI scanner with headphones on. Each participant’s head was restrained with a pad placed behind the neck and between the head and the coil. We asked participants to hold their heads still in the scanner and focus on a fixation cross on a screen. All auditory stimuli were provided both ears through the headphones.

Auditory stimulation was presented using a 4-min-block-design paradigm with 8 blocks of 15 s of rest (stimulation OFF) and 15 s of stimulation including 15 click trains (stimulation ON). Overall, 120 click trains were presented for each fMRI session. Four fMRI sessions were conducted using different sound stimuli for each participant. The stimuli were 1-ms clicks, presented to both ears as trains of clicks for each frequency (80, 40, 30, and 20 Hz). The duration of the click trains and the inter-train interval were both 500 ms, and the click trains were presented at an intensity of 80 dB sound pressure level. We counterbalanced the order of the sessions across participants. Incidentally, the reason for using such a loud sound intensity is that participants hardly hear stimulus sounds at the volume of natural speaking (generally around 60 dB) in the loud noise generated by the MRI scanner.

### Data acquisition

Magnetic resonance imaging was performed using a Philips 1.5-T scanner with standard head coil at the National Hospital Organization Hizen Psychiatric Medical Center. Standard sequence parameters were used to obtain functional images, as follows: gradient-echo echo-planar imaging (EPI); repetition time (TR) = 3,000 ms; echo time (TE) = 45 ms; flip angle = 90°; field of view (FOV) = 230 × 230 mm; matrix = 64 × 64; 60 axial slices with a slice thickness of 4 mm with no slice gap. In between functional data trials, a high-resolution T1-weighted 3D anatomical image of each subject was acquired.

### Image processing

We converted raw image DICOM files to the NIFTI format using MRIConvert (Version 2.0, Lewis Center for Neuroimaging, Oregon). Image processing and statistical analyses were conducted using the statistical parametric mapping software SPM12 (Wellcome Department of Cognitive Neurology, London, United Kingdom) with Matlab R2014b (The Math Works Inc., Natick, MA, United States). We excluded the first five volumes of each EPI image run to let the MR signal to reach equilibrium. After all volumes of the functional EPI images were realigned to the first volume of each session to correct for subject motion, the mean functional EPI image was spatially co-registered with the anatomical T1 image. Each co-registered T1-weighted anatomical image was normalized to a standard T1 template image (ICBM 152) defining the Montreal Neurological Institute (MNI) space. Then we applied the parameters of this normalization process to each functional image. The normalized functional images were smoothened with a 3D 8 mm full-width half-maximum (FWHM) Gaussian Kernel. Time series data for each voxel were temporally filtered using a high-pass filter with a cutoff set to 128 s.

### Statistical analysis

One-way analysis of variance (ANOVA), chi-square test, and *t*-test were used to evaluate group differences in the demographic variables.

The fMRI statistical analysis was performed on the preprocessed EPI using a general linear model (GLM) with a two-level approach (50). The model consisted of boxcar functions convolved with the canonical hemodynamic response function, which was used as the regressor in the regression analysis. In order to reduce motion-related artifacts, six head motion parameters derived from the realignment process were also used as regressors. On the first level of analysis, individual contrast images for each stimulus versus rest were calculated and taken to the second level to make inferences about random effects. On the second level of analysis, contrast images for stimuli as the within-subject factors were submitted to two groups (HC, BD) as the between-subject factors full-factorial ANOVA. All fMRI results are reported at a significance level of *p* < 0.05, family-wise error (FWE)-corrected (voxel-level corrected), or *p* < 0.05, FWE cluster-corrected across the whole brain with the initial voxel threshold at *p* < 0.001, uncorrected. We extracted contrast values by identifying the primary auditory cortex and Brodmann areas 41 and 42 as regions of interest (ROI)s using MarsBar^1^ (Accessed January 3, 2021) to determine the direction of the frequency-by-group interaction. These regions were chosen because ASSR has been reported to be evoked in or near the primary auditory cortex (51, 52). The resulting contrast values were subjected to *t*-tests in SPSS (ver. 28) with the two groups (HC, BD) for each ROI.

## Results

The following is a sequential presentation of the analysis of the subjects’ demographic data, the results of the analysis of ASSR-BOLD, and the relationships between ASSR-BOLD and demographioc/clinical measurements.

### Demographics

We found no significant group differences in the demographic data except socioeconomic status (SES).

### Mean ASSR-BOLD

#### Main effect of group and stimulation

Any significant group differences were not revealed by our full-factorial ANOVA. The main effect of frequency was shown to be associated with significant bilateral activity in Brodmann areas 41 and 42 [left: (−52, −19, 5), cluster size = 5,147, *T* = 11.10, FWE corrected *p* < 0.001; right: (52, −10, 3), cluster size = 5,836, *T* = 9.02, FWE corrected *p* < 0.001] [the (x, y, z) locations are listed in Montreal Neurological Institute coordinates]. Therefore, we focused the rest of analyses on the ASSR-BOLD in Brodmann areas 41 and 42.

#### ASSR-BOLD contrast in broadman areas 41 and 42

**FIGURE 2:**
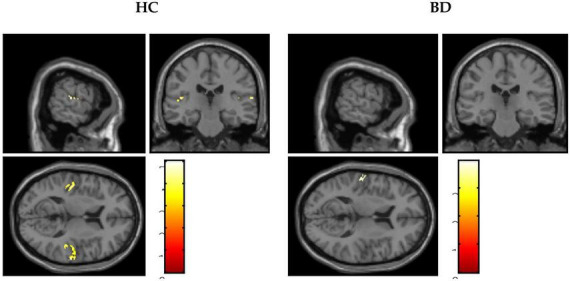
Blood oxygen level-dependent (BOLD) signals activated by 40-Hz click stimuli in the healthy controls (HC) group and the bipolar disorder (BD) group compared with the resting states.

**FIGURE 3:**
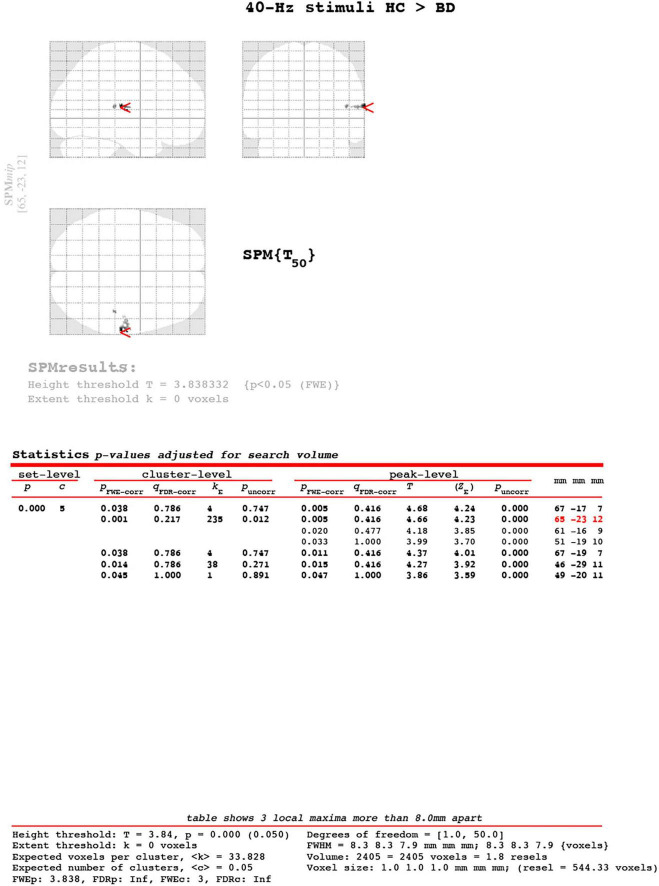
Cluster-level of ASSR-BOLD contrast HC group > BD group for 40-Hz stimuli using SPM 12 defined regions of interest as the right Brodmann areas 41 and 42. The main cluster located [65, −23, 12], the cluster-level was *kE* = 235, pFWE-corrected = 0.001. The Expected voxel per cluster was < k > = 33.828. The [x, y, z] locations are listed in Montreal Neurological Institute coordinates.

**FIGURE 4:**
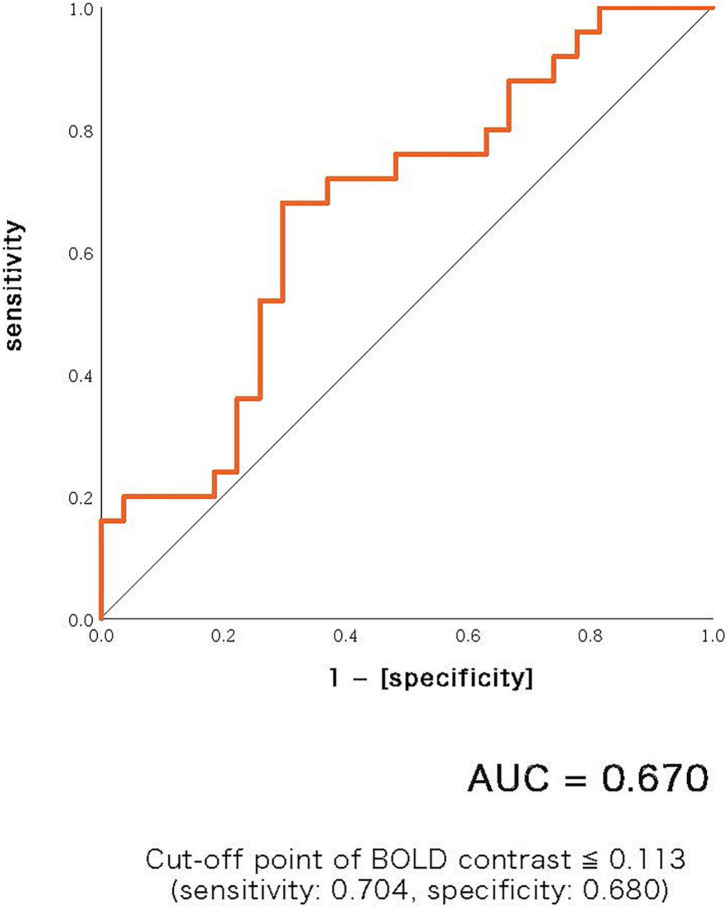
Receiver operating characteristic (ROC) curve showing the ability to discriminate whether a person has bipolar disorder or not by the contrast values of ASSR-BOLD for 40-Hz stimuli in the right Brodmann areas 41 and 42. Area under the curve (AUC) was 0.67. Cut-off point was determined by Youden’s index.

A scattergram of the contrast values of ASSR-BOLD for each stimulus in the right Brodmann areas 41 and 42 is shown in Figure 1. Repeated-measures ANOVAs revealed a significant group difference for 40-Hz stimuli [*F*(1,50) = 5.02, *p* = 0.029], but not 80-Hz stimuli [*F*(1,50) = 0.083, *p* = 0.77], 30-Hz [*F*(1,50) = 0.19, *p* = 0.66] or 20-Hz [*F*(1,50) = 2.95, *p* = 0.092]. On the other hand, there were no significant group differences for each stimulus including 40-Hz in the left Brodmann areas 41 and 42. From these results, it was indicated that ASSR-BOLD signals in response to 40-Hz stimuli were significantly decreased in the BD group in the right Brodmann areas 41 and 42 compared to the HC group in the right Brodmann areas, while there were no significant group differences for 80, 30, or 20-Hz stimuli. We note that the statistical results were essentially the same when SES and Wechsler Adult Intelligence Scale-Third Edition (WAIS-III) information subscale was used as covariates. Comparison of the contrast values of ASSR-BOLD for 40-Hz stimuli compared to the resting state in the right Brodmann areas 41 and 42 in the HC and the BD group is shown in Table 2. Figure 2 shows the BOLD signals activated by 40-Hz click stimuli in the HC and BD groups compared to the resting state. Figure 3 shows cluster-level of ASSR-BOLD contrast the HC group > the BD group for 40-Hz stimuli using SPM 12 defined ROI as the right Brodmann areas 41 and 42. Receiver operating characteristic (ROC) curve (Figure 4) shows the ability to discriminate whether a person has BD or not by the contrast values of ASSR-BOLD for 40-Hz stimuli in the right Brodmann areas 41 and 42. Area under the curve (AUC) was 0.67.

**TABLE 2:** Comparison of the contrast values of ASSR-BOLD for 40-Hz stimuli compared with the resting state in the right Brodmann areas 41 and 42 in the healthy controls (HC) group and the bipolar disorder (BD) group.

	mean	max	min	SD
HC	0.1423	0.4717	−0.2014	0.1906
BD	0.0213	0.3801	−0.5529	0.1987

### Correlations between the ASSR-BOLD and demographic/clinical measurements

**FIGURE 5:**
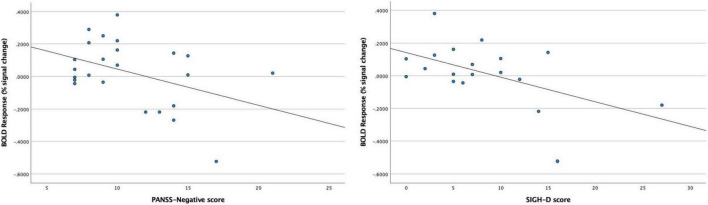
Significant negative correlations between blood oxygen level-dependent (BOLD) response to 40-Hz stimuli in the right Brodmann areas 41 and 42 and PANSS-Negative scores (rho = –0.413, *p* = 0.045) in the BD patients, and between the BOLD response to 40-Hz stimuli in the right Brodmann areas 41 and 42 and SIGH-D scores (rho = –0.530, *p* = 0.020) in the BD patients.

In the BD patients, we found significant negative correlations between the BOLD response to 40-Hz stimuli in the right Brodmann areas 41 and 42 and PANSS-Negative scores (rho = −0.413, *p* = 0.045), and also between the BOLD response to 40-Hz stimuli in the right Brodmann areas 41 and 42 and SIGH-D scores (rho = −0.530, *p* = 0.020) (Figure 5). We found no significant correlations between the BOLD response in the right Brodmann areas 41 and 42 and the other demographic/clinical measurements in the BD patients.

## Discussion

In this study, we compared the ASSR-BOLD elicited by 80-, 40-, 30-, and 20-Hz click trains in BD and HC groups. BD patients showed significantly decreased ASSR-BOLD signals to 40-Hz stimuli compared to HC in the right primary auditory cortex.

Difference in the function of beta and gamma oscillations has been pointed out. Beta oscillations are associated with sensory gating, perception and attention, while gamma oscillations are associated with consciousness and memory as well as perception and attention (1). As mentioned in the introduction, it has been proposed that dysfunction of GABAergic and NMDA receptor is implicated in the pathophysiology of BD (15–20). At the cellular level, the mechanism for the generation and maintenance of gamma oscillations at the cellular level relies on a network of fast-spiking parvalbumin-expressing GABAergic interneurons (8). Furthermore, NMDA receptor signaling in parvalbumin-expressing GABAergic interneurons is essential for the regulation of spontaneous (non-stimulus locked) and evoked gamma oscillations (53). Cunningham et al. (54) suggested that the fast rhythmic bursting neurons in layer II/III were crucial for the generation of gamma oscillations. Traub et al. (55) reported that GABAergic neurons played an essential role in the primary generation of gamma oscillations and their local synchronization. Moreover, even direct electrotonic coupling through gap junctions between inhibitory neurons is involved in the synchronization of gamma oscillations (56). Although high-gamma and low-gamma band oscillations are both produced by repetitive inhibition, their relationship to spiking activity of parvalbumin-containing interneurons differs in terms of profile of pharmacological modulation as much as layer specificity (2). On the other hand, *in vitro* studies have suggested that beta2 (between 20 and 30 Hz) oscillations are generated in a different manner than gamma oscillations. For instance, Roopun et al. (57) reported that beta2 oscillations occurred in layer V pyramidal cells in an *in vitro* study. Furthermore, this study suggested that beta2 oscillations were involved in gap junction coupling and are independent of chemical synaptic transmission. In the present study, it was showed that the gamma-band (40-Hz) ASSR-BOLD was decreased and the beta-band ASSR-BOLD was not significantly decreased in BD patients, indicating that BD could be characterized by functional decline of GABA interneurons associated with fast rhythmic bursting neurons in layer II/III.

As mentioned in the introduction section, it has been reported that BD patients show reduced spontaneous gamma band ASSR in many previous MEG and EEG study, and fMRI can detect evoked and spontaneous gamma activities during auditory stimulation. The present results show that BD patients during 40-Hz auditory stimulation show an overall decrease in neural activation in the right auditory cortex. Then, the question arises as to why there are differences only in the right Brodmann areas 41 and 42, but not in the left Brodmann areas 41 and 42, and why there are differences only for 40-Hz stimulation. Reite et al. (3) investigated ASSR source locations in patient with BD. While for control subjects the right source was superior to the left, no such hemispheric asymmetry was observed in patients with BD. Based on their study, it is likely that stronger differences in BOLD responses occurred in the right Brodmann areas 41 and 42 than in the left Brodmann areas 41 and 42. In addition, it was reported that the presence of a right hemisphere disturbance in BD is consistent with the hypothesis that the right hemisphere may be dominant in mood regulation (58). It is well known that the ASSR is strongest for 40-Hz stimuli in healthy people, thus differences for 40 Hz stimuli between the HC and the BD group would been more likely to observed significantly. It is noted that the latest meta-analysis study provided consistent evidence that 40-Hz ASSRs are reduced in patients with BD compared with HCs (59).

The present results also showed significant negative correlations between 40-Hz ASSR-BOLD responses in the right Broadman areas 41 and 42 and SIGH-D scores; 40-Hz ASSR-BOLD responses in the right Broadman areas 41 and 42 and PANSS-Negative scores, indicating that BD patients with more severe depressive symptoms exhibited more decreased 40-Hz ASSR-BOLD responses in the right Broadman areas 41 and 42.

Considering clinical use, we believe that the biomarkers shown in this study using 1.5-T MRI scanner, which is installed in many facilities, are very useful and will be easier to gather findings at multiple facilities in the future, rather than MEG or research EEG, which are not installed in many facilities.

In comparison with major depression disorder (MDD), Isomura et al. (60) reported that MDD patients exhibited no significant differences in ASSR power compared to HC subjects, while BD patients showed deficits on the gamma band ASSR measures. BD patients showed smaller ASSR power for 40-Hz stimuli compared to MDD patients. This may be reflected in ASSR-BOLD. Future ASSR-BOLD studies should compare BD, MDD, and HC in three groups.

There are several limitations that need to be considered in this study. First of all, not only gamma band activities involve the ASSR-BOLD responses, but also broadband activities involve it. However, it is only gamma band oscillations that are highly correlated with hemodynamics (33). The 40-Hz stimulation might be a frequency that resonates with respect to activation of the ASSR-BOLD signals. In future studies, simultaneous EEG-fMRI recordings should be used for closer examination. Second, the sample size we analyzed in the current study was relatively small (25 BD and 27 HC). Third, we were not able to exclude any treatment effects of antipsychotics, mood stabilizers, antidepressants or benzodiazepines on ASSR-BOLD abnormalities in BD patients. One meta-analysis study suggested that, for bipolar disorder, GABA levels were diminished in medication-free patients, but seemed to be normalized in medicated patients, compared with the healthy controls (61). In EEG study, Rass et al. (25) recorded 50-, 40-, 30-, and 20-Hz ASSRs in BD, and evaluated the ASSR in bipolar disorder and examined its sensitivity to clinical symptoms, cognitive function, and pharmacological treatment. Their study reported that BD patients taking psychotropic medication showed a decrease in phase-locking factor (PLF) compared to BD patients who had ceased medication. Future studies that incorporate pre- and post-medication patient assessments are needed to clarify the relationship between clinical symptoms and ASSR-BOLD deficits in BD patients. Fourth, the AUC shown in Figure 4 was 0.67 which was relatively small. For examples of other biomarkers in psychiatry, anti-TRANK1 IgG assay in SZ yielded AUC of 0.68 (62), and data-driven fusion of brain imaging phenotypes including cortical thickness, surface area and gray matter density maps using linked independent component analysis to identify distinct brain morphology patterns in BD yielded AUC of 0.67 (63). The reliability of 40-Hz ASSR-BOLD in BD shown in this study was slightly inferior or comparable to those markers.

## Conclusion

Overall, in the current study, BD patients exhibited decreased ASSR-BOLD responses in the gamma band, it might be related to an imbalance between GABAergic and NMDA receptor-mediated neurotransmission. In conclusion, the observed decrease in BOLD signal patterns in the right primary auditory cortex during 40-Hz ASSR with 1.5-T MRI scanner in widespread use may be a potential biomarker option for bipolar disorder.

## Data availability statement

The raw data supporting the conclusions of this article will be made available by the authors, without undue reservation.

## Ethics statement

The studies involving human participants were reviewed and approved by The Ethics Committee of National Hospital Organization, Hizen Psychiatric Medical Center. The patients/participants provided their written informed consent to participate in this study.

## Author contributions

HO: conceptualization, methodology, software, validation, formal analysis, investigation, data curation, writing—original draft, review, and editing, and visualization. TO: conceptualization, methodology, software, validation, resources, writing—review and editing, supervision, and funding acquisition. HK: conceptualization, methodology, software, investigation, data curation, resources, visualization, and funding acquisition. NO: methodology, validation, data curation, writing—review and editing, and supervision. NN: investigation and data curation. SF: software. TN: writing—review and editing and supervision. TU: conceptualization, methodology, validation, resources, writing—review and editing, supervision, project administration, and funding acquisition. All authors contributed to the article and approved the submitted version.

## Footnotes

1
http://marsbar.sourceforge.net
